# Tailoring an evidence-based clinical intervention and training package for the treatment and prevention of comorbid heavy drinking and depression in middle-income country settings: the development of the SCALA toolkit in Latin America

**DOI:** 10.1080/16549716.2022.2080344

**Published:** 2022-07-22

**Authors:** Amy O’Donnell, Peter Anderson, Christiane Schmidt, Fleur Braddick, Hugo Lopez-Pelayo, Juliana Mejía-Trujillo, Guillermina Natera, Miriam Arroyo, Natalia Bautista, Marina Piazza, Ines V. Bustamante, Daša Kokole, Katherine Jackson, Eva Jane-Llopis, Antoni Gual, Bernd Schulte

**Affiliations:** a Population Health Sciences Institute, Newcastle University, Newcastle upon Tyne, UK; b Department of Health Promotion, CAPHRI Care and Public Health Research Institute, Maastricht University, Maastricht, The Netherlands; c Centre of Interdisciplinary Addiction Research (ZIS), Department of Psychiatry and Psychotherapy, University Medical Center Hamburg-Eppendorf, Hamburg, Germany; d Addictions Unit, Psychiatry Department, Hospital Clínic, Barcelona, Spain; e Red de Trastornos Adictivos, Instituto Carlos III. Sinesio Delgado, Madrid, Spain; f Institut d’Investigacions Biomèdiques August Pi Sunyer (IDIBAPS); Rosselló, Barcelona, Spain; g Corporación Nuevos Rumbos, Bogotá, Colombia; h Instituto Nacional de Psiquiatría Ram´on de la Fuente, Ciudad de México, Mexico; i School of Public Health and Administration, Universidad Peruana Cayetano Heredia, San Martin de Porres, Peru; j Institute for Mental Health Policy Research, Toronto, Ontario, Canada; k Univ. Ramon Llull, ESADE, Barcelona, Spain

**Keywords:** Alcohol use, depression, cultural adaption, latin america, primary care

## Abstract

**Background:**

Effective interventions exist for heavy drinking and depression but to date there has been limited translation into routine practice in global health systems. This evidence-to-practice gap is particularly evident in low- and middle-income countries. The international SCALA project (Scale-up of Prevention and Management of Alcohol Use Disorders and Comorbid Depression in Latin America) sought to test the impact of multilevel implementation strategies on rates of primary health care-based measurement of alcohol consumption and identification of depression in Colombia, Mexico, and Peru.

**Objective:**

To describe the process of development and cultural adaptation of the clinical intervention and training package.

**Methods:**

We drew on Barrero and Castro’s four-stage cultural adaption model: 1) information gathering, 2) preliminary adaption, 3) preliminary adaption tests, and 4) adaption refinement. The Tailored Implementation in Chronic Diseases checklist helped us identify potential factors that could affect implementation, with local stakeholder groups established to support the tailoring process, as per the Institute for Healthcare Improvement’s Going to Scale Framework.

**Results:**

In Stage 1, international best practice guidelines for preventing heavy drinking and depression, and intelligence on the local implementation context, were synthesised to provide an outline clinical intervention and training package. In Stage 2, feedback was gathered from local stakeholders and materials refined accordingly. These materials were piloted with local trainers in Stage 3, leading to further refinements including developing additional tools to support delivery in busy primary care settings. Stage 4 comprised further adaptions in response to real-world implementation, a period that coincided with the onset of the COVID-19 pandemic, including translating the intervention and training package for online delivery, and higher priority for depression screening in the clinical pathway.

**Conclusion:**

Our experience highlights the importance of meaningful engagement with local communities, alongside the need for continuous tailoring and adaptation, and collaborative decision-making.

## Background

Alcohol and depression make a substantial contribution to the global non-communicable disease burden [1,2]. Alcohol causes three million deaths a year worldwide [3], and according to the World Health Organisation (WHO), depression represents the single largest factor contributing to disability [4]. There is also a strong reciprocal relationship between alcohol use disorders (AUD) and major depression [5–8]. Although data suggest higher rates of depressive disorders are diagnosed in populations living in wealthier regions [9], depression also poses a substantial public health challenge for low- and middle-income countries (LMICs) [10,11]. Likewise, epidemiological studies highlight the high alcohol-attributable disease burden experienced in the global south [3]. In Latin America and the Caribbean, for example, the amount of alcohol consumed per capita is the second highest in the world [12], and it experiences particularly high rates of alcohol-attributable traffic injury including violence [13,14]. As a result, there have been numerous calls to implement more effective and cost-effective policies on alcohol and mental ill-health in this region [15,16].

Effective interventions exist for heavy drinking and depression, including behavioural therapy delivered in primary care settings [17–20], but have seen limited translation into routine practice in global health systems [21]. This evidence-to-practice gap is particularly evident in LMICs [22], with low numbers of appropriate specialist health professionals, limited resources for mental healthcare, and fragmented health systems, highlighted as key barriers to implementation [11,23–25]. Given that the majority of individuals with depressive symptoms are first seen in primary health care (PHC) [26], and based on the aforementioned high comorbidity with AUD [27], helping PHC providers to more efficiently identify and support heavy drinking patients with depression could help address the mental health treatment gap in LMICs [28–30]. In doing so, previous research highlights the importance of providing comprehensive training [31–33], clear, evidence-based clinical materials [34,35], and more supportive community and municipal environments [36–38]. To date, however, with few exceptions (e.g. the WHO Mental Health Gap Action Programme (mhGAP) [39]), most clinical materials and training packages to support the implementation of alcohol and mental health interventions in PHC have been developed and tested in high-income country settings. Numerous studies highlight the need to consider the local cultural and social context when designing and delivering health interventions, especially where psychological interventions are concerned [40–42]. Ecologically relevant, tailored interventions are more likely to be acceptable to patients, more feasible for healthcare providers to deliver, and ultimately have more impact [43–46].

Cultural adaption, or local tailoring, can be defined as *‘the systematic modification of an evidence-based treatment or intervention protocol to consider language, culture, and context in such a way that it is compatible with the client’s cultural patterns, meanings, and values’* [47]. Various approaches have been employed under the label of tailoring (cultural adaption), categorised by Ramaiya (2017) into three main models: 1) non-empirical, in which adaptations are made on an ad-hoc and instinctive basis, without defined elements to preserve fidelity or programme identity; 2) systematic, with explicit conceptual frameworks that suggest when and how interventions should be modified; and 3) stage models that take a middle ground approach and retain fidelity to the original intervention while adapting to culture and context [48]. The latter category, cultural adaption stage models, recognise that when adapting interventions that already have a strong evidence base, there is a need to take the core components from the original intervention as a starting point, before using qualitative approaches in a systematic way to inform and guide adaption into different cultural contexts [49–53]. In doing so, Barrero and Castro (2006) propose a four-stage sequential model, comprising 1) information gathering, 2) preliminary adaption design, 3) preliminary adaption tests, and 4) adaption refinement [54].

Tailoring can also be viewed in terms of efforts to modify clinical materials and delivery strategies to address pre-identified implementation barriers and facilitators. Theory-based frameworks can provide a structure to help researchers assess the potential determinants of implementing new interventions and adapt content and delivery accordingly [55]. The Tailored Implementation in Chronic Diseases framework (TICD) [56] groups these factors (or determinants of practice) into seven overarching domains: guideline factors; health professional factors; patient factors; professional interactions; incentives and resources; capacity for organisational change; and social, political, and legal factors ([57]). The TICD checklist can be used as a screening tool by researchers to identify determinants that warrant further in-depth investigation and can also support the design of appropriate strategies to boost implementation. However, to achieve sustained adoption, The Institute for Healthcare Improvement’s ‘Going to Full-Scale Framework’ stresses the need for supplementary tailoring during the implementation process, alongside introduction of evidence-based adoption mechanisms and support systems, to boost engagement and ownership amongst local stakeholders, and develop appropriate infrastructure [58].

**Figure 1.**
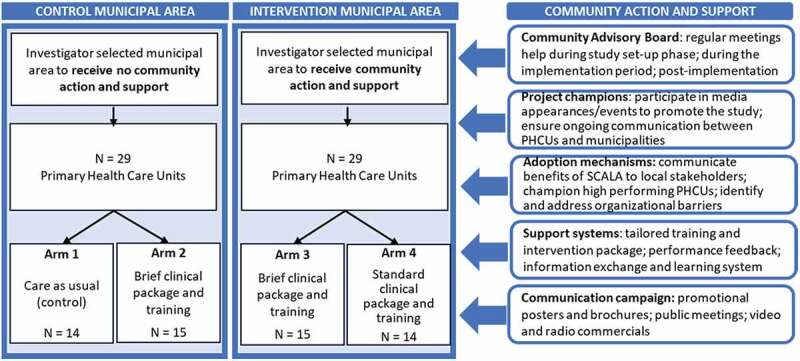
SCALA overall study design.

The international SCALA project (Scale-up of Prevention and Management of Alcohol Use Disorders and Comorbid Depression in Latin America, www.scalaproject.eu) was a quasi-experimental study that sought to test the impact of multilevel implementation strategies on rates of PHC-based measurement of alcohol consumption and identification of depression in Colombia, Mexico, and Peru. The research team comprised international experts in the fields of alcohol prevention and mental health, training and evaluation specialists, implementation scientists, and Latin American academics with links to relevant policymakers, practitioners, and community advocate groups in each country. Full details of the wider study design are provided in our published protocols [59,60] and summarized in Figure 1 below.

In brief, one intervention municipal area was investigator-selected from each of Bogotá (Colombia), Mexico City (Mexico), and Callao–Lima (Peru). One control municipal area was investigator-selected from each of the same cities, based on comparability in terms of socio-economic characteristics, and with sufficient geographical separation to minimize spill-over effects from the intervention municipal area. 58 PHC units (PHCUs) were recruited across these municipal areas. Half of these PHCUs (29) were in municipal areas which did not benefit from additional community support measures. Of these, 14 PHCUs comprised our control (Arm 1) and 15 PHCUs received a brief clinical intervention and provider training package alone (Arm 2). The remaining 29 PHCUs received additional community action and support measures with 15 PHCUs randomly allocated to receive a brief (less intensive) tailored clinical intervention and provider training package (Arm 3) and 14 PHCUs to receive a standard (more intensive) version of this package (Arm 4). The primary outcome was the cumulative proportion of consulting adult patients out of the population registered within the participating PHCUs whose alcohol consumption was measured during the study period (i.e. coverage).

The community action and support measures employed in Arms 3 and 4 were based on the Institute for Healthcare Improvement Going to Scale Framework [58] and consisted of five core blocks of activity designed to encourage PHC providers to deliver brief alcohol and depression advice to their patients (see Figure 1). First, the creation of local stakeholder groups (Community Advisory Boards) to advise on tailoring, support implementation, and review drivers of successful action. Second, the appointment of local project champions to advocate for successful implementation in each intervention municipality. Third, implementation of evidence-based adoption mechanisms focused on actions by the local research team and project champion to communicate the advantages of and need for the SCALA program with relevant municipal stakeholders. Fourth, implementation of evidence-based support systems, including the development of training and intervention packages tailored to the needs of local PHC providers and patients, and provision of regular performance review feedback and an interactive information exchange and learning system for participating PHC providers. Fifth and finally, implementation of community-based communication campaigns, including dissemination of posters and leaflets about the study in relevant spaces, plus the use of promotional videos and radio appearances.

In this paper, we describe the process of development and cultural adaption of the SCALA clinical intervention and training package which was a key component of the multi-level implementation strategies outlined above. In doing so, we seek to provide a worked example of how a multi-national research team can draw on available tailoring and adaption frameworks to support the design and implementation of a culturally appropriate public health intervention in Latin America.

## Methods

### Implementation context

Colombia, Mexico, and Peru are all classed as upper-middle-income countries [61,62], but the structure and capacity of their respective PHC systems vary. In recent years, Colombia and Mexico in particular have sought to strengthen PHC provision and boost population access to healthcare (e.g. Colombia’s 2016 Comprehensive Health Care Model (Modelo Integral de Atención en Salud, MIAS) and Mexico’s 2015 Comprehensive Health Care model (MAI)) [63,64]. However, high turnover in primary care personnel, and the related impacts on continuity of information and care coherence, continue to prove challenging [65]. Whilst average primary care appointments are relatively lengthy (average 15 minutes in Peru compared to 10 minutes in the UK (UK) [66]), limited time is available for professional training and development. Existing practice around alcohol and mental health also varies. In Peru, there are no explicit guidelines, although healthcare providers are encouraged to include alcohol as part of mental health-related screening activities [67]. In Colombia, recommendations around alcohol screening and brief interventions are included as part of clinical practice guidelines focussed on detection and treatment of alcohol abuse and dependence in healthcare, however no official standards exist [68]. In contrast, Mexico has implemented standards stipulating the process and criteria for the mandatory prevention, treatment, and control of addictions, which include asking questions about alcohol use [69] and documenting this information in patient records [70].

### Approach to tailoring and adaption

The overarching ethos informing the SCALA tailoring process most closely represented the cultural adaptation stage model; i.e. middle-ground between a universal (or top-down), and a culture-specific (or bottom-up) approach [48]. As such, we sought to incorporate necessary modifications to improve cultural fit whilst maintaining core intervention components where robust evidence of their effectiveness existed. In doing so, we drew on Barrero and Castro’s four-stage sequential model [54], using the TICD checklist to identify potential factors that could affect the adoption and use of the intervention package [57]. Additionally, as per the Institute for Healthcare Improvement’s Going to Scale Framework, we also created local stakeholder groups to support the tailoring process [58].

Implementation factors from each of the seven TICD domains (guideline factors, individual health professional factors, patient factors, professional interactions, incentives, and resources, capacity for organisational change, and social, political, and legal factors) were reviewed and rated by the research team according to their relevance for this specific project (see supplementary materials). Certain guideline factors were rated as not requiring further action due to the strength of existing international evidence demonstrating their effectiveness (e.g. [17]). Additionally, no action was recommended in relation to some social, political and legal factors as they were deemed to be fundamentally outside the control of the study team. All other factors were incorporated in the tailoring process and described according to their importance and urgency for action.

**Figure 2.**
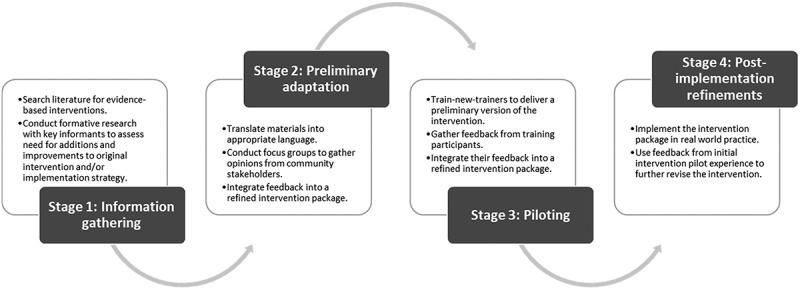
Stages of SCALA cultural adaptation process.

In stage one, we drew on existing empirical evidence, theory, and international best practice guidelines to produce outline versions of all clinical and training materials. Next, in stage two, these outline versions were scrutinised by Latin American stakeholders (policymakers, academics, practitioners and patients) and adapted into locally tailored prototype versions that could be implemented and evaluated in stages three and four. Stage three comprised piloting the full intervention package at ‘train-new-trainers’ sessions for trainers from all participating municipalities held in Bogota, Colombia, and incorporating further refinements in response to their feedback. Finally, in stage four, this refined version of the package was implemented in routine clinical practice in participating municipalities, with continuous feedback captured to inform any subsequent changes required. See Figure 2 below.

## Results

### Stage 1: information gathering

We planned to use guidelines for the prevention and treatment of heavy drinking and depression from each Latin American country as the starting point for developing the clinical intervention package. However whilst some relevant clinical guidelines existed (e.g. Mexican standards for the prevention, treatment, and control of addictions [69], and alcohol screening and brief intervention recommendations in Colombia [68]), there had been limited translation of these guidelines into user-friendly materials for clinical providers in any participating country. Likewise, we were unable to identify well-established local initiatives providing appropriate provider training on alcohol or mental health interventions. Therefore, we based initial drafts of the clinical materials on relevant international best practice and evidence, drawing in particular on the UK National Institute for Health and Clinical Excellence Guidelines for hazardous and harmful drinking prevention [71]. For the training package, content drew on the WHO alcohol brief intervention training manual for primary care [72] and the PHEPA (Primary Health Care European Project on Alcohol) Training Programme on Identification and Brief Interventions [73].

Next, we gathered additional data on contextual factors that needed to be considered when developing the intervention and training package via formative discussions with key local informants based in our target municipalities. These data included: 1) definitions of a standard alcoholic drink and official guidelines on ‘lower risk’ drinking for each country; 2) local specialist service provision for alcohol and mental health, and 3) ability of PHC providers and trainers to carry out alcohol and mental health prevention work. Some key issues highlighted during this process concerned the limited skills and capacity of trainers in the target municipalities, and the restricted opportunities for specialist mental health or alcohol treatment referrals from PHC [74]. We also identified a particular need for training to help boost self-efficacy amongst PHC providers in relation to alcohol identification practices across the three participating countries [75]. The output from this stage was a proposed structure and outline content for the intervention and training package, in English and Spanish, respectively, including: a top-level clinical protocol; more detailed guidance for PHC providers; patient information leaflets on heavy drinking and depression; an outline training manual for trainers; and materials for use in train-new-trainer sessions.

### Stage 2: preliminary adaptation

Initial versions of all clinical materials were translated into Spanish and, along with the training package materials, were shared with relevant municipal stakeholders for discussion and feedback, i.e. local study partners and CAB members. Where needed, additional ad-hoc meetings with local government and clinical representative organisations took place, e.g. in Colombia, meetings were held with the Health Secretary of the Government of Cundinamarca (state in which both intervention and control municipalities were located). As well as providing general feedback on the content and structure of the intervention and training package, stakeholders also suggested priority health or social issues they felt important to highlight in these materials. For example, in Peru, CAB members suggested highlighting alcohol-related violence; in Colombia, members wanted to emphasize the causal link between alcohol and various cancers. Stakeholders in all three countries also helped identify relevant contact details that could be added to the clinical materials to help signpost patients to appropriate specialist services or information sources.

**Figure 3.**
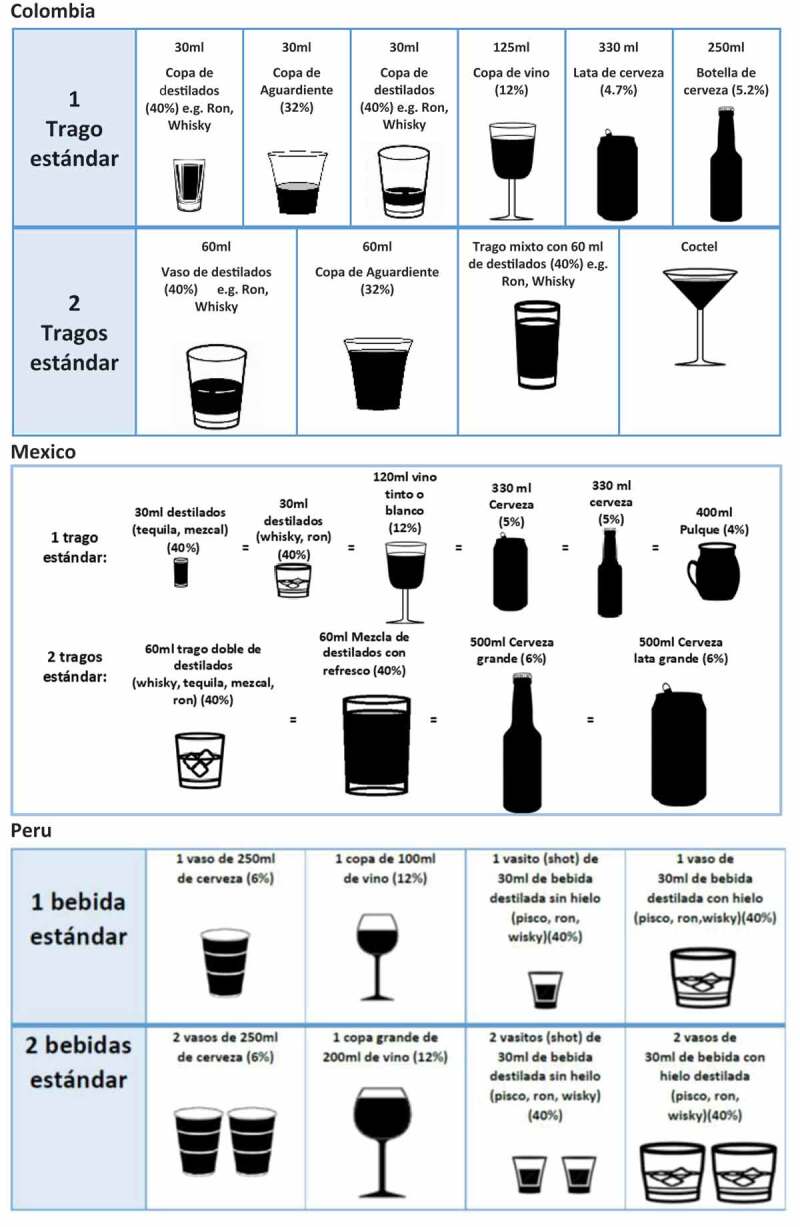
Tailored standard drinks tables for Colombia, Mexico and Peru.

Next, we gathered qualitative feedback on all intervention materials via facilitated discussions with Patient and Clinical Provider User Panels established in each municipality. For the alcohol information leaflets, User Panel discussions focused on the need to translate some core concepts and terminology to better suit the local implementation context. For example, Patient User Panel members in Bogota, Colombia found the advice about what constituted a standard alcoholic drink confusing and requested more relevant examples of typical Colombian drinks adding to the leaflets, an issue which was also raised by the Provider User Panel in Peru. To address these concerns, we developed a set of images of common local drinks for each country to help illustrate what we meant by a ‘standard drink’. As well as referencing appropriate local alcoholic beverages, e.g. Tequila in Mexico and Pisco in Peru, the imagery also sought to show the different types of drinking vessels used for certain drinks in each country (see Figure 3).

**Figure 4.**
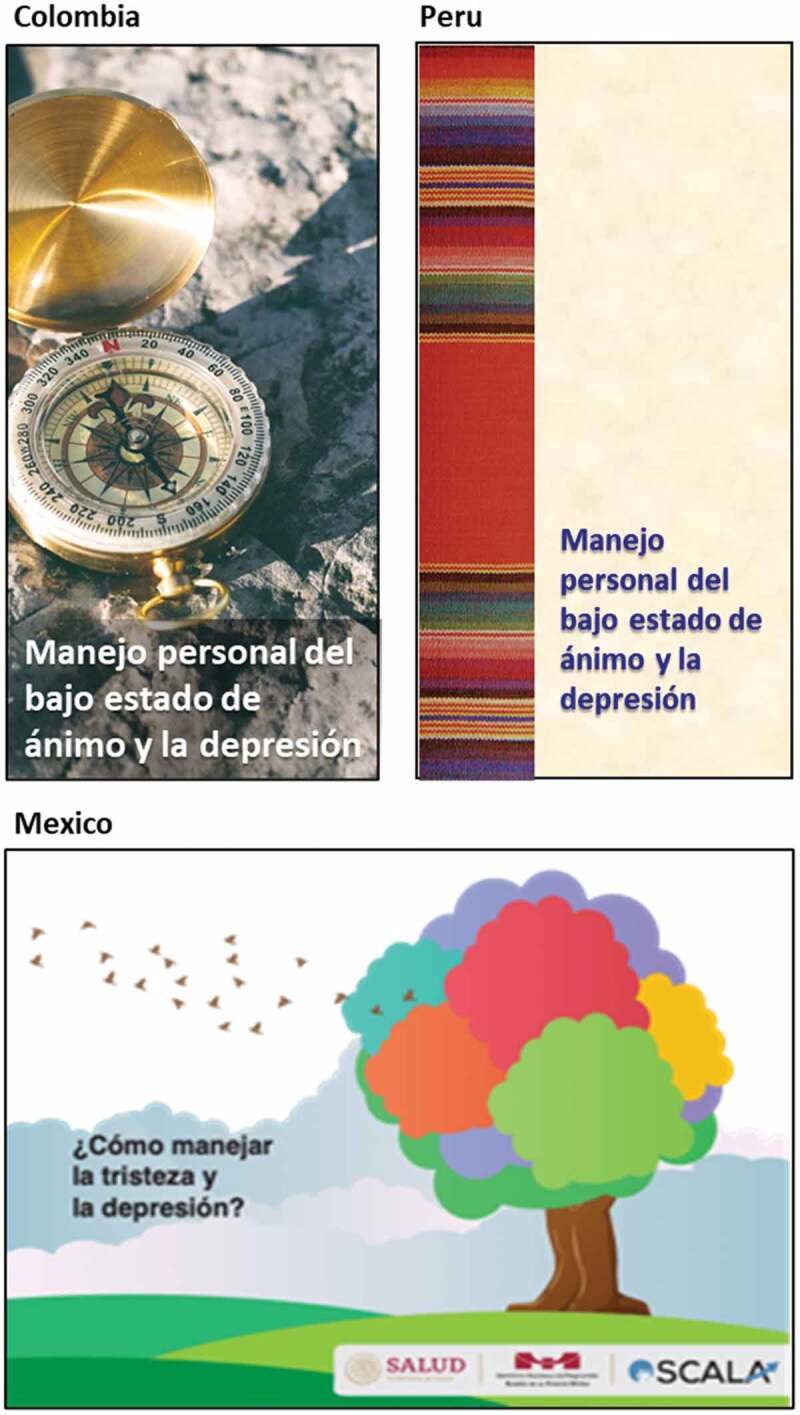
Style and presentation of patient information leaflets for depression.

For the depression information leaflets, User Panel feedback highlighted a need to adapt the visual presentation of materials to reflect local culture, values, and social norms. For example, each country selected different images to embody key health promotion messages; advice to ‘*try and keep occupied with activities you generally enjoy*’ was represented by a guitar for Colombia, by a guitar and a book for Peru, and by gardening imagery for Mexico. Additionally, in tailoring the overall ‘look’ of the leaflet, Peru chose to use colours and imagery based on recognisably Peruvian ethnic textiles. In Mexico, stakeholders wanted to use neutral imagery to avoid reproducing negative stereotypes associated with depression (see Figure 4).

The initial draft of the training manual was reviewed by key informants in each country including local health professionals responsible for implementing new clinical protocols in daily practice. Reflecting the above User Panel feedback, informants stressed the need to tailor examples of common drinking situations appropriately, using the typical beverage consumed typical pattern of consumption in each country. Additionally, although all training materials had been translated into Spanish, differences emerged in how certain words were used or defined in European Spanish compared to each Latin American country. For example, the word for screening is ‘cribado’ in European Spanish, however ‘tamizaje’ is used in Latin-America. Differences were also identified in the words used to represent key terms between Latin America partner countries. For example, the word for ‘referral’ in Mexico and Peru is ‘referencia’, whereas ‘remisión’ is used in Colombia. Scripts for use in the intervention modelling videos were developed through live role-play exercises by the lead clinical professionals on the Spanish training team, then transcribed and translated for assessment by the Latin America project partners. Feedback stressed the need to reflect regional accents in the videos to be used in the training sessions, and to involve actors from the three participating countries. Finally, discussions highlighted a need to boost capacity in training provision in each country via implementation of a training-new-trainers (TNT) course [76,77].

As we moved towards finalising the materials in October 2018, concerns were raised by Latin American partners regarding the length and complexity of the proposed intervention package as likely implementation barriers. We therefore decided to develop a short, less intensive version of the clinical intervention package, and to amend the research design to enable us to test the impact of both the standard and short-form versions on implementation rates. Therefore, the final output from this stage was a set of locally tailored clinical intervention and training materials, including modelling videos, and clinical intervention materials in short and long forms that could be subsequently piloted with local trainers during a TNT course.

### Stage 3: piloting in training-new-trainers course

The TNT course took place in Bogota, Colombia in May 2019, and was led by a native-Spanish speaking addiction specialist with experience of both implementing brief interventions in PHC and training clinicians. Over two days, future trainers from Colombia, Mexico, and Peru were given the opportunity to experience the training and practice its components, including using the various clinical materials, followed by reflexive discussion sessions where participants could make suggestions for further improvements. As a result, a few minor additional refinements to the clinical materials were suggested, mostly to ensure consistency across the intervention package. Participants also suggested developing some new materials to support implementation in busy PHC practice: a study desk sign for clinicians to prompt patients to initiate a conversation about heavy drinking and/or depression during appointments, and a pocket-sized version of the clinical protocol that clinicians could carry around during practice.

Additionally, participants requested we develop some practical tools to help local trainers to deliver the training package in their respective municipality, e.g. a checklist detailing key logistical issues to consider when organising local training sessions, such as ensuring suitable equipment is available to project the intervention modelling videos. Further, as no follow-up TNT sessions were planned due to budgetary constraints, it was also suggested that we worked with participants to produce a series of videos that could be shared on social media to highlight key take-home messages from the training. Participants also requested that we establish ‘real-time’ communication channels between the local trainers and the study team, via a dedicated a *WhatsApp* group. This would allow us to continue to adapt the intervention to specific local contexts (accents, dialect) and unexpected events (lack of internet access), helping to maintain the pace and relevance of training sessions.

### Stage 4: post-implementation refinements

The final stage comprised further refinements in response to feedback from Latin American study partners, CAB members and PHC providers following ‘real-world’ delivery of the SCALA package. Importantly, this included a period of implementation under relatively ‘normal’ conditions (August 2019-March 2020), followed by the onset of the COVID-19 pandemic. In the initial pre-COVID implementation period, refinements focussed on measures to ensure the clinical intervention and training package was sufficiently agile and responsive to the needs of the local healthcare context. From April 2020, more substantive changes were needed to sustain implementation following the rapid onset of COVID-19 which resulted in significant restrictions to daily life and public services in Colombia, Mexico, and Peru, particularly healthcare. The combination of COVID-related pressures and reduced PHC access required us to ‘pause’ implementation in Colombia and Peru in March 2020. In Mexico, there was no official ‘pause’ and some PHC providers continued to use the clinical materials throughout.

Subsequently, as the unprecedented scale and impact of the pandemic became apparent over the course of 2020, we recognised that even if it was possible to resume implementation, modifications would be needed to adapt the package to the changing landscape of global PHC, and to ensure we could efficiently train any new providers that had joined the PHCUs during the ‘pause’ period. This resulted in two key additional adaptations. First, we wanted to build on opportunities provided by the rapid move to telehealth in global healthcare during COVID-19 [78]. Our training package was therefore adapted for online delivery, meaning it could be accessed remotely, and repeatedly, at no additional cost. In Peru specifically, the intervention package was also translated for online implementation, automating the clinical care pathway, and facilitating access to electronic versions of the patient materials. This was not possible in Colombia and Mexico, primarily due to lack of internet connection in the PHCUs. Second, we recognised the substantial adverse impact the pandemic and related lockdown measures were having on mental health in Latin America and elsewhere [79]. In response, for providers, we developed an extra training package session focusing on mental health and resilience amongst PHC clinicians. For patients, we amended the clinical pathway, to give increased priority to depression screening and care in the intervention package. This meant that all consulting patients would be screened for both depression and heavy drinking.

Additionally, the decision was made to drop the use of the standard (more intensive) package previously implemented in Arm 4, in favour of the brief (less intensive) alternative used in Arm 3. This was partially based on feedback from local providers responsible for implementing the package, who reported that they found the standard version excessively time-consuming and thus impractical for delivery in routine practice. We also determined through analysis of interim trial outcome data (alcohol measurement and depression identification rates) that there was no significant difference in performance between the two arms, meaning that we could not justify continued use of the standard package on implementation-effectiveness grounds [80].

## Final package

Following these four stages of adaption, the final SCALA clinical intervention and training package included the following elements. A **refined clinical care pathway** specifying how alcohol and depression screening, intervention delivery and/or referral procedures should be implemented in routine consultations in PHC. The final version required providers to screen all consulting adult patients for both heavy drinking and depression, and for Peru only, was translated for online delivery. **Culturally adapted clinical materials** comprising: 1) validated alcohol measurement and depression screening instruments (three question Alcohol Use Disorder Identification Test-Consumption (AUDIT-C); two question Patient Health Questionnaire (PHQ-2) [81,82]; 2) brief patient advice material for those identified with heavy drinking and/or depression and a positive re-enforcement leaflet for lower risk drinkers; 3) provider guidelines (detailed and ‘pocket-sized’ form); and 4) study desk sign for clinicians. As above, materials 1, 2 and 3 were translated for online use in Peru only. A **locally tailored training package** consisting of four products: 1) a training manual and annexes including evaluation questionnaires and hand-outs; 2) examples of common clinical situations recorded in short modelling videos with professional actors from Peru, Colombia, and Mexico; 3) training course presentations, and 4) additional training-new-trainer materials including the two-day in-person course and follow-up ‘reminder’ videos. All the aforementioned materials are available via the SCALA study website in English (Project outputs (scalaproject.eu)) and Spanish (Resultados del proyecto (scalaproject.eu)).

## Discussion

This paper describes our efforts to systematically tailor and adapt a clinical and training package to address heavy drinking and depression in Latin American PHC as part of the international SCALA study. In doing so, we provide a worked example of how existing tailoring and implementation frameworks, specifically the TICD checklist [57] and Institute for Healthcare Improvement Going to Scale framework [58], can support us to adapt evidence-based interventions to better fit specific local cultural contexts and health priorities. The process of adaption itself broadly followed Barrero and Castro’s four stage sequential model [54], beginning with an initial phase of information gathering local contextual intelligence, through successive cycles of refinement and improvement, including changes made in response to feedback provided following implementation in real-world practice. The pace and scale of adaptation was of course substantially affected by the impact of COVID-19 on global healthcare systems from March 2020 onwards, resulting in a rapid move to online training and in the case of Peru, online clinical intervention delivery. As such, our experience has potential relevance to academics, policymakers and practitioners undertaking cultural adaption of health interventions in a range of circumstances, and specifically, responds to an identified lack of evidence on the experiences of Latin American communities who have been involved in these sorts of initiatives [83].

Efforts to promote meaningful community engagement at each stage comprised a critical element of our adaption process. Multiple stakeholders were involved to ensure that decisions around both the content and style of the intervention and training package, and the implementation strategies employed, were informed by those actually affected by their application [84]. The training package, for example, was informed by a modelling perspective designed to both empower trainers and in turn to raise awareness of the need to empower their trainees [85–87]. This approach aligns with best-practice recommendations for research collaboration governance with LMIC partners [88], and reflects widespread recognition of the value of community and stakeholder engagement in achieving both the instrumental and ethical objectives of health intervention research programmes [89–91]. Additionally, we worked with patients, healthcare providers, and community leaders responsible for delivering and receiving the intervention package, alongside regional and national policymakers with the authority to address system level barriers that could impede sustained implementation. Importantly, these community engagement activities took place in varied spaces in an attempt to limit the potential impact of intrinsic power disparities between different groups of stakeholders, and were conducted throughout the study, meaning we could respond in real-time to challenges and opportunities as they emerged [91]. Previous evidence suggests that tailored interventions are more likely to have positive impact compared to generic approaches [43–46], and confirms the importance of involving community stakeholders in the development and delivery of alcohol prevention programmes [92,93]. Interim results from the wider SCALA study suggest that the tailored training package implemented in our intervention municipalities has led to increased coverage of alcohol measurement, and in turn depression screening, amongst PHC patients [80,94].

Various frameworks informed our approach to implementation and tailoring at the outset of the study, most notably the TICD [57] and Institute for Healthcare Improvement Going to Scale [58]. However, as the research progressed, and, as the scale of the pandemic became apparent, there was a need for greater flexibility and pragmatism on the part of both the research team and the local implementers. As emphasised in Chambers et al’s Dynamic Sustainability Framework (DSF), there is increased understanding that sustained implementation of an intervention requires us to engage in a process of continuous monitoring, negotiation and adapation, with a primary focus on contextual fit [95]. This is particularly the case where complex interventions are concerned (i.e. those that: involve multiple, interacting components, target several providers, settings and health system levels, and implicitly permit a degree of flexibility in how, when and where an intervention is delivered [96]). For example, when we originally designed the clinical care pathway, local partners were reluctant to recommend that PHC providers screened all patients for heavy drinking and depression simultaneously due to concerns that this would result in excessive workloads. However, growing recognition of the adverse impact of COVID-19 on global mental health resulted in the decision to reprioritise identification and support for depression in the pathway, alongside the development of support materials for providers themselves. Achieving such responsiveness required flexibility on the part of the intervention and training development team, and active, ongoing communication between the international study partners and local stakeholders. Online video conferencing platforms (e.g. Microsoft Teams) were essential to support regular international team meetings, a development recognised elsewhere as having the potential to promote more equitable global collaboration in research [97]. Responsiveness also demanded that the (mostly European) research team and (mostly Latin American) local implementers navigate some challenges around the ultimate ownership of and responsibility for the SCALA ‘package’ and reconceptualise ‘intervention’ drift or deviation from protocol as a legitimate and necessary stage in the implementation process. As such, this approach reflects the recent, comprehensive definition of sustainability in healthcare innovation proposed by Moore, which acknowledges that an intervention or programme may need to evolve or adapt in order to ensure continued benefits for individuals, care providers, and systems [98]. However, our study had a few limitations. Notably, the onset of the COVID-19 pandemic meant we were unable to implement planned community support initiatives that we hoped would help provide a more supportive implementation context, and work to shift local cultural attitudes towards alcohol and mental health. This potentially explains why interim results from the wider study found no evidence to support the added value of these initiatives on rates of alcohol measurement in participating PHCUs [80]. Bernal and others have highlighted the importance of addressing the wider context in which the patient is immersed as part of the adaption process, particularly in the case of behaviors and practices that are intrinsically shaped by culture and social norms [50,54,99]. As tailoring of the clinical and training package comprised just one component of the multilevel implementation strategy being tested in the wider SCALA study, we are unable to quantify the extent to which cultural adaptation alone contributed to improvements in alcohol and/or depression screening rates. Future research could compare the impact of a tailored versus standard clinical package on intervention delivery, as well as exploring the specific contribution of community participation, through an appropriately designed study [100]. Additionally, there were influencing factors beyond the control of the research team that would have also affected implementation. Early in the adaptation process, in our TCID assessment of contextual barriers and facilitators, we made a conscious decision not to devise implementation strategies explicitly focussed on factors that would require national-level legislative change to address. As others have written, one key limitation of the TICD is that it is not always feasible to address certain determinants of practice through research studies [56]. Finally, whilst international research partnerships provide invaluable opportunities for the exchange of skills, expertise, and cross-cultural learning, particularly around addressing mental ill-health [101], we must also acknowledge the inherent challenges for teams that span multiple geographic, temporal, and socio-cultural boundaries, including language differences. Throughout our study, we actively sought to build constructive and meaningful relationships between partners, via regular in-person and online meetings. However, again, the impact of COVID-19 on international travel, and research and health system workloads, undoubtedly made this more difficult as our study progressed. We hope to explore these issues in future planned outputs from the wider SCALA project, in particular, those from the process evaluation conducted alongside the main quasi-experimental study [59].

## Conclusion

This paper describes the process of developing and adapting a clinical intervention and training package to address heavy drinking and depression for implementation in Colombia, Mexico, and Peru. In doing so, we seek to add to the scant evidence on structured approaches to tailor evidence-based practices to meet the needs of different global mental health and substance use contexts. Our experience highlights the importance of meaningful engagement with local communities, alongside the need for continuous tailoring and adaptation, and collaborative decision-making. Future research could help further unpack the impact of cultural adaptation on implementation outcomes.

## Supplementary Material

Supplemental Material

## Paper context

Effective interventions exist for heavy drinking and depression but there has been limited translation into routine practice in global health systems. We tested the impact of multilevel implementation strategies, including the development of a culturally adapted clinical intervention and training package, on the rates of primary health care-based measurement of alcohol consumption and the identification of depression in Colombia, Mexico and Peru. Our experience highlights the importance of meaningful engagement with local communities, alongside the need for continuous tailoring and adaptation, and collaborative decision-making.
