# Supplementary material for: Tailoring an evidence-based clinical intervention and training package for the treatment and prevention of comorbid heavy drinking and depression in middle-income country settings: the development of the SCALA toolkit in Latin America
Source: Glob Health Action. 2022 Jul 22;15(1):2080344. doi: 10.1080/16549716.2022.2080344 (PMC9310809; doi:10.1080/16549716.2022.2080344)
Supplement: Supplemental Material [file ZGHA_A_2080344_SM0688.docx]

## Appendix: Mapping SCALA implementation determinants and strategies against the TICD Checklist

| **Key:** |  | No action required^[[1]](#footnote-2)^ |  | Important and urgent^[[2]](#footnote-3)^ |  | Important but less urgent^[[3]](#footnote-4)^ |  | Less important and not urgent^[[4]](#footnote-5)^ |  | Not relevant^[[5]](#footnote-6)^ |
| --- | --- | --- | --- | --- | --- | --- | --- | --- | --- | --- |

| DOMAIN | Definition | Response | ACTION |
| --- | --- | --- | --- |
| 1. GUIDELINE FACTORS | | | |
| Recommendation | | | |
| **Quality of evidence supporting the recommendation** | How confident we are in the estimates of effects | Moderate-high quality RCTs, assessed in multiple robust systematic reviews. A specific SCALA adoption mechanism is demonstration of the alignment of the SCALA package with the latest evidence of preventing and managing AUD and comorbid depression. | N/A |
| **Strength of recommendation** | How confident we are that the desirable effects of adherence to the recommendation outweigh the undesirable effects | Latest Cochrane review (Kaner 2018) confirms small but significant impact of brief interventions for alcohol on weekly alcohol consumption. As above, a specific SCALA adoption mechanism is demonstration of the alignment of the SCALA package with the latest evidence of preventing and managing AUD and comorbid depression. | N/A |
| **Clarity** | The clearness of the target population, the settings in which the recommendation is to be used and the recommended action | The protocol stipulates a defined municipal population in each country partner.  The action will be primarily delivered via primary health care units and supported by a detailed protocol for healthcare professionals. A specific SCALA adoption mechanism is demonstration of the superiority and simplicity of the SCALA package. | N/A |
| **Cultural appropriateness** | The extent to which recommendation is suitable in the social context where it is being implemented | SCALA was informed by understanding of epidemiological need for study (in terms of prevalence of AUD/depression in Colombia, Mexico, Peru). Various adaptations were made to the care pathway/protocol on the basis of regular discussions with representatives from each country level partner. | - Feedback on protocol needed from Patient and Provider UPs to ensure focus takes into account local social context. For example, issues of stigma, drinking culture etc. - Research team to:   - Refine final version of protocol and associated materials as needed.   - Develop media campaigns to boost alcohol health literacy etc based on CAB/UP feedback. - Process evaluation will also gather further empirical data to assess social / cultural acceptability in local municipal social contexts. |
| Accessibility of the recommendation | How accessible the guideline or recommendation is | Various adaptations have already been made to the accessibility of the care pathway / protocol on the basis of: advice from LA partners; and advice from health literacy expert. | - LA partners to forward / backward translate the protocol. - Critical review via UPs to assess accessibility of language / format of materials and suggest any refinements required. - Possible options include provision in variety of formats, electronic and hard-copy - Research team to refine final version as needed. - Process evaluation will also gather further empirical data to assess accessibility in local municipal social contexts. |
| Source of the recommendation | The organisation(s) and people that made the recommendation | Materials drew on range of credible sources including international WHO and UK NICE guidelines. SCALA materials to be ‘branded’ with endorsement of local organisations as relevant.  In addition, the establishment of the CABs will also help promote endorsement by a range of relevant organisations and individuals (also see above re adoption mechanism) | N/A |
| Consistency with other guidelines | The extent to which recommendation is consistent with recommendations in other guidelines with which the targeted healthcare professionals might be familiar | Consistency checks have been carried out during calls with LA partners as part of the development of all materials alongside desk-based work to identify any other existing policy guidelines. | - Discussions at CABs to evaluate fit with existing municipal guidelines for alcohol/mental health care. - CABs also to review for fit within local municipal context and suggest any adaptations - Discussions should focus in particularly on identifying any additional specialist service provision in area and / or highlighting specific areas of concern. - Research team to address any discrepancies, examine reasons for any conflicts, and refine materials accordingly. For example, some conflicts may be unavoidable but will be important to explain reasons why conflicts exist. |
| Recommended clinical intervention | | | |
| Feasibility | The extent to which the recommended clinical intervention is practical | Feasibility considerations informed development of study and materials. This included availability of specialist alcohol/mental health services in each municipality. | - Details to be incorporated within protocols   Process evaluation to gather further empirical data to inform adaption / further roll-out of intervention package outside immediate municipalities. |
| Accessibility of the intervention | The extent to which the recommended clinical intervention is accessible | A clear protocol including recommended care pathway will be provided to all municipalities (intervention and comparator cities). Various elements of the pathway (cut-off scores in particular) have been purposefully simplified to promote easy adoption. | N/A |
| Recommended behaviour | | | |
| Compatibility | The extent to which the recommended behaviour fits with current practices | Discussions held with LA partners to explore any potential conflict with PHC practice at municipal level.  Training package to provide strategies to support providers to incorporate intervention into daily practice | - CABs to monitor implementation and support providers where incompatibilities arise or communicate with research team where adjustments are needed   Process evaluation to gather further empirical data to inform adaption / further roll-out of intervention |
| Effort | The amount of effort required to change or adhere | LA partners have advised on this point but potential issues when implemented at PHCU level | - CABs to monitor adherence issues and support providers where incompatibilities arise or communicate with research team where adjustments are needed, for example to simplify process or highlight where additional assistance/training is needed.   Process evaluation to gather further empirical data to inform adaption / further roll-out of intervention |
| Trialability | The ability to try out the recommended behaviour | Tailored training package includes role-play scenarios to provide supportive context in which providers can try out protocol. | N/A |
| Observability | The degree to which benefits of the recommended behaviour are visible | Communicating impact is likely to improve adherence/engagement in study (and beyond) | - Process and outcomes evaluation to gather further empirical data on impact to inform adaption / further roll-out of intervention - Study team/CABs to provide regular performance feedback to providers throughout study. |
| 2. INDIVIDUAL HEALTH PROFESSIONAL FACTORS | | | |
| Knowledge and skills | | | |
| Domain knowledge | The extent to which the targeted healthcare professionals have pre-existing knowledge or expertise about the targeted condition | Although health systems in each country have responsibilities around prevention/management of AUD/depression, skill-set at PHCU provider level varied/unknown. Comprehensive training package to be implemented at municipal level which includes content aimed at increasing provider knowledge about AUD and depression. | - Feedback from Provider UP needed on full training package. - Process evaluation to monitor training impact |
| Awareness and familiarity with the recommendation | The extent to which the targeted healthcare professionals are aware of and familiar with the recommendation | SCALA will address this via training and provision of clear, locally-tailored care pathway to all PHCUs.  Comprehensive training package will also be implemented at municipal level which includes content aimed at increasing provider awareness of AUD /depression care pathway. Municipal media campaigns will also be used to increase awareness of SCALA. | - Ongoing monitoring/evaluation via CABs to identify any concerns/issues arising at PHCU level. - Potential to use booster training sessions to address any issues highlighted. |
| Knowledge about own practice | The extent to which the targeted healthcare professionals are aware of their own practice in relationship to the recommended practice | Comprehensive training package to be implemented at municipal level. As part of this training, providers will be encouraged to reflect on existing practice and highlight specific areas for development/ adaptation. Monitoring and feedback will also be implemented to address this. | - Ongoing monitoring/evaluation via CABs to identify any concerns/issues arising at PHCU level. - Potential to use booster training sessions to address any issues highlighted. |
| Skills needed to adhere | The extent to which the targeted health professionals have skills that they need to adhere | Comprehensive training package to be implemented at municipal level. As part of this training, providers will be introduced to core skills required to implement the SCALA protocol. | - Ongoing monitoring/evaluation via CABs to identify any concerns/issues arising at PHCU level. - Potential to use booster training sessions to address any issues highlighted. |
| Cognitions | | | |
| Self-efficacy | The targeted healthcare professionals’ self-perceived competence or confidence in their abilities | Comprehensive training package to be implemented at municipal level. SCALA training package is designed to improve self-efficacy in providers | - Ongoing monitoring/evaluation via CABs to identify any concerns/issues arising at PHCU level. - Potential to use booster training sessions to address any issues highlighted. |
| Learning style | The preferred ways in which the targeted healthcare professionals learn | Comprehensive training package to be implemented at municipal level. SCALA training package is designed to incorporate range of learning styles, including interactive/role-play opportunities. | - Ongoing monitoring/evaluation via CABs to identify any concerns/issues arising at PHCU level. - Potential to use booster training sessions to address any issues highlighted. |
| Emotions | The extent to which emotions affect adherence | Comprehensive training package to be implemented at municipal level. As part of this training, providers will have opportunity to discuss potential emotional barriers/facilitators relating to implementing the SCALA protocol. | - Ongoing monitoring/evaluation via CABs to identify any concerns/issues arising at PHCU level. - Potential to use booster training sessions to address any issues highlighted. |
| Professional behaviour | | | |
| Nature of the behaviour | Characteristics of the behaviour, including: frequency of performance for a patient, frequency of performance for a population of patients, the degree of habit or automaticity, whether it is within a sequence of other behaviours that have to be performed, and whether it is performed by one person or by different people | SCALA will implement ongoing audit and feedback processes to assess adoption rates, and support PHCUs to adapt practice infrastructure as necessary.  Booster training sessions will be used to help further embed new practices and professional behaviours. | - Ongoing monitoring/evaluation via CABs to identify any concerns/issues arising at PHCU level. - Potential to use booster training sessions to address any issues highlighted. |
| Capacity to plan change | The extent to which the targeted healthcare professionals have the capacity to plan necessary changes in order to adhere | SCALA will promote the development of appropriate infrastructure for scale-up. Part of these include the provision of tailored protocols (e.g. materials, instruments, checklists, data capture systems etc), alongside making key personnel available to support adoption (e.g., data managers, quality improvement mentors, municipal champions). | - Ongoing monitoring/evaluation via CABs to identify any concerns/issues arising at PHCU level. - Potential to use booster training sessions to address any issues highlighted. |
| Self-monitoring or feedback | The extent to which the targeted healthcare professionals have the capacity for self-monitoring or feedback to reinforce adherence with the recommendation | SCALA will introduce data collection and reporting systems to support PHCUs to track and evaluate performance. The data gathered will be shared frequently with frontline staff and system leaders to inform ongoing improvement. | - Ongoing monitoring/evaluation via CABs to identify any concerns/issues arising at PHCU level. - Potential to use booster training sessions to address any issues highlighted. |
| 3. PATIENT FACTORS | | | |
| Patient needs | Real or perceived needs and demands of the patient | The SCALA intervention package includes tailored materials for patients and providers (alongside training) designed to: raise awareness of patient need around AUD and depression; and reframe views about alcohol. Mass media campaigns will also contribute to this latter aim. | - Process evaluation to assess impact on adoption and public engagement |
| Patient beliefs and knowledge | Patients’ beliefs or knowledge or ability to learn, or the targeted healthcare professionals’ ability or perceived ability to inform or teach patients necessary knowledge and skills | The SCALA intervention package includes tailored materials for patients designed to reframe views, knowledge and awareness about alcohol and depression. Mass media campaigns will also contribute to this latter aim. Additionally, health literacy component will support providers to assess patient capacity to process and apply health information, and implement additional support as needed. | - Feedback on protocol needed from Patient UPs to ensure materials take into account local beliefs/knowledge. - WP2 team to:   - Refine final version of protocol and associated materials as needed.   - Develop media campaigns to boost alcohol health literacy etc based on CAB/UP feedback. - Process evaluation will also gather further empirical data to assess social / cultural acceptability in local municipal social contexts. |
| Patient preferences | Patients’ values in relationship to professional values or those in the recommendation | The SCALA intervention package includes tailored materials for patients and providers (alongside training) designed to: raise awareness of patient need around AUD and depression; and reframe views about alcohol. This should improve alignment between these groups in terms of shared values/beliefs around alcohol and depression. | - Process evaluation to assess impact on adoption and public engagement |
| Patient motivation | The targeted healthcare professionals’ ability or perceived ability to motivate patients to adhere | In addition to patient information leaflets, training, and mass media campaigns, health literacy component will support providers to assess patient capacity to process and apply health information, and implement additional support as needed. | - Process evaluation to assess impact on adoption and public engagement |
| Patient behaviour | Patient behaviours that motivate or demotivate adherence with the recommendation | The SCALA training package will focus on providing PHCUs with evidence-based strategies to boost patient adherence to advice on alcohol and depression. The health literacy component will also support providers to understand patient capacity to process and apply health information | - Process evaluation to assess impact on adoption and public engagement |
| 4. PROFESSIONAL INTERACTIONS | | | |
| Communication and influence | The extent to which the targeted healthcare professionals’ adherence is influenced by professional opinions and communication | Via the CABs, SCALA will engage identified leaders and build their capacity to understand the difference between simply raising awareness of better practice and what it takes to lead and ensure broad adoption of the SCALA package through guiding and supporting large-scale change. | - Potential issue for ongoing monitoring and feedback by CABs. - Process evaluation to assess impact on adoption |
| Team processes | The extent to which professional teams or groups have the skills needed to adhere and interact in ways that facilitate or hinder adherence | SCALA will support the development of local team leaders in change management approaches, improve the skills of municipal-based data managers and appoint a local integrator (champion and knowledge and practice broker). | - Potential issue for ongoing monitoring and feedback by CABs. - Process evaluation to assess impact on adoption |
| Referral processes | Processes for transferring patients and communication between different levels of care, between health and social services, and between the targeted healthcare professionals and targeted patients | SCALA will introduce simple, tailored care pathways and associated information materials/contact details/training to facilitate efficient identification, support and referral for patients. | - Potential issue for ongoing monitoring and feedback by CABs. - Process evaluation to assess impact on adoption |
| 5. INCENTIVES AND RESOURCES | | | |
| Availability of necessary resources | The extent to which the resources that are needed to adhere are available | In short term, SCALA funds to support delivery of activity, including training and provision of relevant materials. In medium-longer term, the establishment of CABs is designed to promote sustainability of protocol in municipalities. | - Potential issue for ongoing monitoring and feedback by CABs. - Process evaluation to assess impact on adoption |
| Financial incentives and disincentives | The extent to which patients, individual health professionals and organisations have financial incentives or disincentives to adhere | No direct incentives provided within SCALA additional to those described above. | - Potential issue for ongoing monitoring and feedback by CABs. - Process evaluation to assess impact on adoption |
| Nonfinancial incentives and disincentives | The extent to which patients, individual health professionals and organisations have nonfinancial incentives or disincentives to adhere | Indirect incentives include:   - ecognition of SCALA training package by relevant bodies - Ongoing performance feedback - Implementation of simple, evidence-based protocol for dealing with AUD and depression. | - Potential issue for ongoing monitoring and feedback by CABs. - Process evaluation to assess impact on adoption |
| Information system | The extent to which the information system facilitates or hinders adherence | Although electronic PHC systems are insufficiently mature to permit integration of protocol at present, SCALA will develop infrastructure for scale-up including relevant tools (e.g. checklists, data capture systems) and personnel (e.g., data managers) to support ongoing management of PHCU performance. | - Potential issue for ongoing monitoring and feedback by CABs. - Process evaluation to assess impact on adoption |
| Quality assurance and patient safety systems | The extent to which existing quality assurance or patient safety systems facilitate or hinder adherence | Development of infrastructure for scale-up will include relevant tools (e.g. checklists, data capture systems) and personnel (e.g., data managers) to support ongoing management of quality and safety.  Quality/safety data will be regularly shared with frontline staff and system leaders to inform ongoing improvement. | - Potential issue for ongoing monitoring and feedback by CABs. - Process evaluation to assess impact on adoption |
| Continuing education system | The extent to which the continuing education system facilitates or hinders adherence | SCALA will deliver a comprehensive training package to address any inadequacies of existing CPD provision in municipality. | - Potential issue for ongoing monitoring and feedback by CABs. - Process evaluation to assess impact on adoption |
| Assistance for clinicians | The extent to which clinicians have the assistance they need to adhere | Development of infrastructure for scale-up will include relevant tools (e.g. checklists, data capture systems) and personnel (e.g., data managers) to support ongoing adherence by PHC providers. | - Potential issue for ongoing monitoring and feedback by CABs. - Process evaluation to assess impact on adoption |
| 6. CAPACITY FOR ORGANISATIONAL CHANGE | | | |
| Mandate, authority, accountability | The mandate, authority and accountability for making necessary changes | Purpose of CABs is to gather endorsements from a range of organisations and individuals that are highly relevant to the aims of SCALA. As part of this action, SCALA will also support the development of local team leaders in change management approaches, improve the skills of municipal-based data managers and appoint a local integrator (champion and knowledge and practice broker). | - Potential issue for ongoing monitoring and feedback by CABs. - Process evaluation to assess impact on adoption |
| Capable leadership | The extent to which clinical leaders or managers are capable of making necessary changes | As above, SCALA will support the development of local team leaders in change management approaches, improve the skills of municipal-based data managers and appoint a local integrator (champion and knowledge and practice broker). | - Potential issue for ongoing monitoring and feedback by CABs. - Process evaluation to assess impact on adoption |
| Relative strength of supporters and opponents | The extent of support and opposition to necessary changes | CABs will play a key role in addressing potential opposition by bringing together a range of relevant organisations and individual across the municipality. The appointment of a local integrator (champion and knowledge and practice broker) will also support this aim. | - Potential issue for ongoing monitoring and feedback by CABs. - Process evaluation to assess impact on adoption |
| Regulations, rules, policies | The extent to which organisational regulations, rules or policies facilitate or hinder necessary changes | CABs will play a key role in addressing potential regulatory issues by bringing together a range of relevant organisations and individual across the municipality. The appointment of a local integrator (champion and knowledge and practice broker) will also support this aim. | - Potential issue for ongoing monitoring and feedback by CABs. - Process evaluation to assess impact on adoption |
| Priority of necessary change | The relative priority given to making necessary changes | Development of infrastructure for scale-up will focus on addressing structural needs with *existing* resources. A local integrator (champion and knowledge/practice broker) will be appointed. Responsibilities include brokering shared health goals across the municipality and addressing relevant community resource issues, both of which will also contribute to this aim. | - Potential issue for ongoing monitoring and feedback by CABs. - Process evaluation to assess impact on adoption |
| Monitoring and feedback | The extent to which monitoring and feedback are needed at organisational level and available to sustain necessary changes (including evaluations of improvement programs) | Development of infrastructure for scale-up will include relevant tools (e.g. checklists, data capture systems) and personnel (e.g., data managers) to support ongoing management of PHCU performance. Process/outcome data will be regularly shared with frontline staff and system leaders to inform ongoing improvement. | - Potential issue for ongoing monitoring and feedback by CABs. - Process evaluation to assess impact on adoption |
| Assistance for organisational changes | The extent to which external support is needed and available for necessary changes | CABs will play a key role in addressing potential need for external support for PHCUs by bringing together a range of relevant organisations and individual across the municipality. The appointment of a local integrator (champion and knowledge and practice broker) will also support this aim. SCALA will also boost professional capacity for scale-up, through trained frontline PHCU staff and municipal level teams. | - Potential issue for ongoing monitoring and feedback by CABs. - Process evaluation to assess impact on adoption |
| 7. SOCIAL, POLITICAL AND LEGAL FACTORS | | | |
| Economic constraints on the health care budget | Limits on the total healthcare budget or its growth | SCALA approach emphasises need to implement package within existing resources as much as possible. However substantial cuts to health budgets may adversely affect initial and long-term adoption. | - Potential issue for ongoing monitoring and feedback by CABs - Process evaluation to assess impact on adoption |
| Contracts | The extent to which contracts may affect implementation of necessary changes | Composition and ToRs of CABs have been specifically designed to consider whether any adaptations might be required to support implementation of SCALA intervention package. | - Potential issue for ongoing monitoring and feedback by CABs. - Process evaluation to assess impact on adoption |
| Legislation | The extent to which legislation may affect implementation of necessary changes | Certain legislation/regulations are supportive of the SCALA approach but important to monitor and consider impact of new developments over study timeline | - Potential issue for ongoing monitoring and feedback by CABs. - Process evaluation to assess impact on adoption |
| Payer or funder policies | The extent to which payer or funder policies may affect implementation of necessary changes | Composition and ToRs of CABs have been specifically designed to consider whether any adaptations might be required to support implementation of SCALA intervention package. | - Potential issue for ongoing monitoring and feedback by CAPs. - Process evaluation to assess impact on adoption |
| Malpractice liability | The extent to which malpractice liability may affect implementation of necessary changes | SCALA approach emphasises need for supportive environment incorporating positive performance monitoring/feedback. | - Issue for ongoing monitoring as part of CAB discussions. - Identified concerns to be escalated to SCALA study team as needed. |
| Influential people | The extent to which influential people may affect implementation of necessary changes | Composition of CABs has been specifically designed to engage local opinion leaders in development and implementation of SCALA intervention package.  Additionally, an integrator (champion and knowledge and practice broker) will be appointed in each municipality with additional leadership, facilitation, evaluation etc responsibilities. | - Issue for ongoing monitoring and feedback by local CABs. - Process evaluation to assess impact on adoption |
| Corruption | The extent to which corruption may affect implementation of necessary changes | Robust and transparent monitoring and feedback systems implemented as part of SCALA will boost potential for concerns around corruption to undermine adherence | - Issue for ongoing monitoring as part of CAB discussions. - Identified concerns to be escalated to SCALA study team as needed. |
| Political stability | The extent to which political stability may affect implementation of necessary changes | Engagement of local policymakers and commissioners should strengthen sustainability of SCALA intervention package against political instability but fundamental changes to system will be challenging to manage. | - Issue for ongoing monitoring as part of CAB discussions. - Identified concerns to be escalated to SCALA study team as needed. |

1. Issues already incorporated/addressed within SCALA [↑](#footnote-ref-2)
2. Issues discussed in first round of CAB/UP meetings [↑](#footnote-ref-3)
3. Issues for action/discussion immediately post-implementation of intervention package [↑](#footnote-ref-4)
4. Issues for ongoing monitoring by CABs and process evaluation [↑](#footnote-ref-5)
5. Issues fundamentally outside control of SCALA [↑](#footnote-ref-6)
